# The influence of big five personality traits on anxiety: The chain mediating effect of general self-efficacy and academic burnout

**DOI:** 10.1371/journal.pone.0295118

**Published:** 2024-01-02

**Authors:** Xiaoying Wu, Weina Zhang, Yihui Li, Lange Zheng, Jingyu Liu, Yaye Jiang, Yan Peng

**Affiliations:** 1 School of Psychology and Mental Health, North China University of Science and Technology, Tangshan, Hebei Province, China; 2 School of Social Sciences, Universiti Sains Malaysia, Penang, Malaysia; University of Maribor, SLOVENIA

## Abstract

**Background:**

As an important factor affecting personal health, anxiety has always been valued by people. Prior research has consistently shown that personality traits is associated with anxiety level,but little is known about the inner mechanism of this relationship. To fill the gap, the present research aims to explore the chain mediating role of general self-efficacy and academic burnout in the relationship between big five personality and anxiety.

**Methods:**

This cross-sectional study was performed from September to November 2022. Self-reported questionnaires including the Big Five Personality Questionnaire, General Self-Efficacy Scale, College Student’s academic burnout Scale, Generalized Anxiety Scale and demographic characteristics were distributed to 2505 college students in a university in Hebei Province, of which 2,471 were valid. Statistical analysis was carried out through SPSS26.0 and SPSS PROCESS macro.

**Results:**

Results showed four of the big five personality characters (i.e., extraversion, agreeableness, conscientiousness, and openness) were negatively correlated with anxiety. Neuroticism was positively correlated with anxiety. Moreover, general self-efficacy was found to be negatively correlated with academic burnout and anxiety; academic burnout was positively correlated with anxiety. Finally, general self-efficacy and academic burnout mediated the relationship between personality traits (i.e., extraversion, agreeableness, neuroticism, openness) and anxiety.

**Conclusions:**

Personality traits (i.e., extraversion, agreeableness, neuroticism, and openness) could influence anxiety through the chain mediating effects of general self-efficacy and academic burnout. Interventions focusing on anxiety reduction may be successful in increasing general self-efficacy and decreasing students’ academic burnout.

## Introduction

Mental health problems among college students are becoming increasingly prominent in China, and anxiety is one of them [1]. According to relevant data from the World Health Organization, since 2005, the number of patients with anxiety disorders has increased by 14.9%. In the population of college students, a medium to high level of anxiety has been identified [2]. As one of the most common psychological disorders, anxiety has attracted attention from public health because it will result in negative impacts on health, such as sleep disruption, asthma, heart disease, stroke, and cancer [3–6]. It is of great significance to deeply explore the antecedents of anxiety and its potential inner mechanism for providing information to alleviate anxiety in the population of college students.

Combining insights from the Five-Factor Model, personality traits are enduring patterns of thoughts, emotions, and behaviors with five dimensions: extraversion, agreeableness, conscientiousness, neuroticism, and openness [7]. An extensive amount of research has been shown the association between Big Five personality traits and affective symptoms. For example, Prenoveau et al. [8] conducted a three-year-longitudinal study and found a medium to high level relationship between personality traits and anxiety during adolescence and early adulthood. Alizadeh et al. [3] indicated that neuroticism was positively associated with anxiety disorders, while the other four personality traits were significant predictors of being unaffected by mental health. Several researches have identified a strong association between neuroticism and anxiety levels [9–13]. However, conflicting results are still exist. In a study of Chu et al., he found that conscientiousness positively affects the level of anxiety [9]. While in Nouri’s study,conscientiousness was assumed to be a negative predictor of anxiety [10]. Although abundant researches have been conducted to explore the relationship between Big Five personality and anxiety, few scholars take the perspectives of college students and examine the inner mechanism in this relationship.

College students are the core force that promotes a country’s social progress and development. They are more susceptible to anxiety, depression, and other mental diseases due to changes in social roles, decreased social support and increased pressure [14]. Consequently spearking, it is of great practical significance to explore the relationship between different personality traits and anxiety among college students. Personality is a relatively stable individual difference, and it is challenging to reduce anxiety symptoms by changing personality traits [15,16]. Prior studies have shown that personality and emotion are related through coping behavior [17]. For example, Taylor proposed in the hierarchical model that personality traits serve as high-level susceptibility factors for anxiety, the mediating role played by more specific secondary factors should also be considered [18]. In this study, we aim to explore the inner mechanism of the association between personality traits and anxiety and provide information for improving mental health among college students in China.

General self-efficacy refers to the degree of self-confidence individuals have about their ability to achieve specific achievements through their abilities [19]. Personality traits are part of core self-evaluation and self-concept, whereas general self-efficacy is one of its components [20]. As an essential internal psychological resource of individuals, self-efficacy has a strong relationship with the Big Five personality. Evidence suggested that a lower level of neuroticism and a higher level of extraversion, openness, agreeableness, and conscientiousness could lead to a higher level of general self-efficacy and individuals with higher general self-efficacy may have a decreased level of anxiety [21–24]. According to the Social Cognitive Theory, self-efficacy is an intermediary variable linking personality traits and social behavior [25]. Meanwhile, according to Bandura, the lack of belief in the ability someone has to solve problems may affect a person’s neuroticism and will finally result in anxiety [26]. Therefore, we have some reasons to believe that self-efficacy plays a part in the relationship between personality traits and anxiety, so we put forward Hypothesis 1: Self-efficacy plays a mediating role between the Big Five personality and anxiety.

Other evidence suggests that academic burnout is another potential mediator. West defined burnout as a syndrome of emotional exhaustion, depersonalization, and low personal accomplishment that culminates in decreased effectiveness at work [27]. Academic burnout was applied when studying burnout in the population of students [27,28]. In the ecological model of human development, the developing individual is at the center of the ecosystem and interacts with the surrounding environmental systems to influence the development of the individual [29]. A meta-analysis examining the relationship between five personality traits and academic achievement has demonstrated that conscientiousness and academic achievement were most strongly associated, followed by openness and agreeableness, and neuroticism and extraversion were generally weakly or not associated with academic achievement [30]. Personality characteristics can also be expressed through learning styles (auditory, visual, reading/writing, kinesthetic). These can help students better understand and study to achieve ideal learning effects and goals [31]. When students achieve the academic goals they set, their sense of academic accomplishment will increase, and their sense of academic burnout will decrease and finally alleviate. Therefore, we have reason to believe that there is a specific correlation between personality traits and academic burnout, and at present, there are studies to support this conjecture [32]. It is inevitable to feel academic burnout and mental disorder for college students when facing too much academic stress, thus resulting in anxiety. Based on Social Identity theory, individuals with high academic accomplishment may have a more positive view of themselves [33]. On the contrary, it is hard to maintain a positive social identity in the face of low academic status [34]. It can be seen that academic achievement greatly affects the anxiety of college students. In addition, the conservation model of resources is based on the supposition that people strive to retain, protect, and build resources to protect themselves from potential or actual threats. According to this model, burnout, as a consequence of stress, continuously consumes the emotional and cognitive resources, which can protect the individual from new stressors from outside, thus creating psychological symptoms, such as anxiety [35]. Until now, the association between burnout and anxiety has already been examined [36]. Therefore, we proposed Hypothesis 2: academic burnout mediating the relationship between Big Five personality and anxiety.

At the same time, Bandura proposed reciprocal causal links exist between person, environment, and behavior [19]. From this point of view, we can infer that the environment could affect the mental health among college students; And college students can change the situation or adjust themselves to the environment [37]. Personality represents an internal trait, while self-efficacy refers to a cognitive structure that explicitly expresses an individual’s internal characteristics, so it plays a different role in affecting academic achievement [38]. Schunk found self-efficacy played a role in the learning process. It can enhance students’ learning motivation [39]. In return, when students experience a high level of learning motivation, they maintain a good sense of self-efficacy. An experiment constructed in 2020 supports the idea that motivation is negatively correlated with burnout [40,41]. Specifically speaking, the enhancement of students’ learning motivation implies the feeling of academic burnout, which in turn alleviates the negative emotion caused by burnout. Related research on general self-efficacy and academic burnout found that general self-efficacy and academic burnout were negatively correlated [42]. To sum up, there is a joint contribution of general self-efficacy and academic burnout in the relationship between personality traits and anxiety. Therefore, we put forward Hypothesis 3: general self-efficacy and academic burnout play a chain mediating role between the big five personality traits and anxiety.

The current research investigates the combined effect of general self-efficacy and academic burnout on the relationship between the Big Five personality and anxiety to provide theoretical guidance for alleviating anxiety symptoms and improving mental health situations among college students with different personalities.

## Methods

### Participants

This study used a convenience sampling method to conduct a questionnaire survey among students at a university in Hebei Province. A total of 2,505 questionnaires were distributed, of which 2,471 were valid. The effective questionnaire return rate was 98.64%. Among them were 1094 males and 1386 females, aged 17–29, with an average age of 19.65±0.03. Exclusion criteria for invalid questionnaires: the questionnaire’s content is incomplete, the answers are regular, the data has extreme values, and the answering time is too short. Participation in this study is solely voluntary. Participants who gave their informed consent in the survey were directed to answer the questions upon their agreement. This study has been approved by the Ethics Committee of North China University of Science and Technology (No.2021037).

### Procedure

The questionnaire of this study was posted online to Questionnaire Star platform (https://www.wjx.cn/) for recruit potential participants. Questionnaire Star platform is an online platform that allows researchers to post self-administered surveys to recruit participants, and it is widely accessible by the public. We distributed the QR code of the questionnaires in the campus forum online and invited the participants to participate. To obtain the written informed consent of the participants, we briefly explained the purpose of the questionnaire to every participant before s/he started to answer. Participants’ right to withdraw was informed and there was no honorarium provided for answering the survey.

Participants filled up the demographics (i.e., gender, age, grade, place of birth). Followed by completing the Big Five Personality,General Self-Efficacy Scale, College Students’ academic burnout and lastly the Generalized Anxiety Scale. At the end of the questionnaire, participants were thanked for their participation. Each participant took approximately 15 to 20 minutes to answer the questionnaire. Their participation was anonymous, with no incentive given upon completion. All participants were given the option to withdraw at any moment.

### Materials

*Big Five Personality Questionnaire* The scale includes five dimensions: Extraversion, Agreeableness, Conscientiousness, Neuroticism, and Openness [43]. A total of 60 items, using the Likert 5-point scoring method. The Cronbach’s α coefficient of the scale was 0.78.

*General Self-Efficacy Scale* The scale is used to measure the general self-efficacy of the subjects [44]. The Likert 4-point scale has ten items in total. Higher scores represent higher levels of general self-efficacy. The Cronbach’s α coefficient of the scale in this study was 0.95.

*College Students’ academic burnout Scale* The scale is used to evaluate the academic burnout of college students [45]. The scale has 20 items in total with a Likert 5-level scoring method. In this study, Cronbach’s α coefficient of the scale was 0.81.

*Generalized Anxiety Scale* The scale is used to assess the anxiety symptoms of the subjects [46]. Seven items were on the scale, and the Likert 4-level scoring method was used. The higher the score, the more severe the anxiety symptoms of the subjects. In this study, Cronbach’s alpha coefficient of the scale was 0.94.

### Statistical analyze

SPSS 26.0 was used for the common method bias test, descriptive statistics analysis, variance analysis, and correlation analysis. Mediation model testing was performed using model 6 of the PROCESS program developed by Hayes.

Independent sample t-tests were used to analyze gender differences in personality, general self-efficacy, academic burnout, and anxiety among the subjects. F-test were used to determine differences in personality, general self-efficacy, academic burnout, and anxiety level by grade.

Pearson’s correlation test was used to examine the bivariate correlation between the variables (i.e.,Big Five personality, general self-efficacy, academic burnout and anxiety).

Hypothesis 1(Self-efficacy plays a mediating role between the Big Five personality and anxiety) and Hypothesis 2 (academic burnout mediating the relationship between Big Five personality and anxiety) were tested by performing the mediation test on the PROCESS macro program of SPSS26.0 plug-in.

As for the Hypothesis 3 (general self-efficacy and academic burnout play a chain mediating role between the big five personality traits and anxiety), it was tested by having Extraversion, Agreeableness, Conscientiousness, Neuroticism and Openness as independent variables, anxiety as dependent variables, and general self-efficacy and academic burnout as mediators in running the mediation test according to Bootstrap. Gender, grade and place of birth were acted as controlled variables to construct a qualified mediation model. Significance level of 0.05 was used to identify significant results.

## Results

### Common method bias test

Harman’s single factor test was performed on the data to test for common method bias. The results showed that 14 factors existed higher than 1, and the variance explained by the first factor was 21%, less than the critical standard of 40%, indicating no apparent common method bias.

### Differences in big five personality demographic variables, general self-efficacy, academic burnout, and anxiety

Agreeableness and conscientiousness were significantly associated with academic burnout in the population of college students of different genders. There were statistically significant differences in the scores of agreeableness, conscientiousness, and academic burnout between college students of different genders (*P*<0.05), and there were significant differences in the scores of extraversion, agreeableness, neuroticism, and anxiety among college students of different grades. Statistically significant (*P*<0.05), there is a statistically significant difference in openness scores between college students from different places of origin (*P*<0.05). See Table 1.

**Table 1:** Analysis of variance of demographic variables.

Item	Number	Extraversion	Agreeableness	Conscientiousness	Neuroticism	Openness	General self-efficacy	Academic burnout	Anxiety
Gender									
Male	1087	36.35±5.22	40.74±6.28	39.51±6.47	33.74±6.23	38.68±5.62	28.26±6.15	57.67±8.97	10.62±4.34
Female	1384	36.45±5.91	43.14±6.32	40.47±6.24	33.72±6.68	39.41±5.90	28.13±4.75	56.78±9.09	10.68±3.97
*t*		-0.426	-9.413	-3.710	0.094	-3.121	0.584	2.446	-0.368
*P*		0.670	<0.001	<0.001	0.925	0.002	0.559	0.015	0.713
Grade									
Freshman	1447	36.48±5.89	42.53±6.42	40.38±6.46	33.33±6.67	39.31±5.92	28.28±5.37	57.03±9.33	10.57±4.13
Sophomore	421	36.93±4.65	40.35±6.22	39.34±6.00	33.86±5.80	38.64±5.20	28.06±5.92	57.53±8.55	10.13±4.12
Junior	564	35.90±5.54	42.18±6.42	39.72±6.37	34.58±6.45	38.91±5.91	28.06±5.19	57.30±8.70	11.15±4.08
Senior	39	35.18±5.33	42.77±4.90	40.08±5.37	34.54±5.83	38.51±5.25	27.95±4.35	56.38±8.76	11.85±4.29
*F*		3.468	12.963	3.569	5.321	1.829	0.331	0.473	6.327
*P*		0.016	<0.001	0.014	0.001	0.140	0.803	0.701	<0.001
Place of birth									
City	786	37.07±6.11		42.36±6.79	33.25±6.65	40.11±6.41	28.53±5.84	56.56±9.62	10.51±4.06
Countryside	1685	36.10±5.35	41.96±6.23	39.89±6.14	33.95±6.39	38.62±5.41	28.02±5.20	57.45±8.76	10.72±4.17
*t*		3.843	1.401	1.674	-2.467	5.653	2.059	-2.208	-1.194
*P*		<0.001	0.162	0.094	0.014	<0.001	0.040	0.027	0.233

### Correlation analysis of big five personality, general self-efficacy, academic burnout, and anxiety

Extraversion (36.41±5.61), agreeableness (42.07±6.41), conscientiousness (40.05±6.36), and openness (39.09±5.79) were positively correlated with general self-efficacy (28.17±5.44) and academic burnout (57.18±5.44) 9.04), but negatively correlated with anxiety (10.65±4.15). Neuroticism (33.71±6.48) was negatively correlated with general self-efficacy but positively correlated with academic burnout and anxiety. General self-efficacy was negatively correlated with academic burnout and anxiety. Academic burnout is positively correlated with anxiety. See Table 2.

**Table 2:** Correlation of each scale (r-value, n = 2480).

**Item**	**Gender**	**Grade**	**Place of birth**	**Extraversion**	**Agreeableness**	**Conscientiousness**	**Neuroticism**	**Openness**	**General self-efficacy**	**Academic burnout**	**Anxiety**
Gender	1										
Grade	0.02	1									
Place of birth	0.05*	-0.07*	1								
Extraversion	0.001	-0.04*	-0.08**	1							
Agreeableness	0.19**	-0.04	-0.03	0.37**	1						
Conscientiousness	0.07**	-0.05*	-0.04	0.48**	0.69**	1					
Neuroticism	-0.002	0.08**	0.05*	-0.53**	-0.59**	-0.63*	1				
Openness	0.06**	-0.04	-0.12**	0.48**	0.52**	0.55*	-0.44**	1			
General self-efficacy	-0.01	-0.02	-0.04*	0.32**	0.29**	0.36*	-0.33**	0.33**	1		
Academic burnout	-0.05*	0.01	0.05	-0.38**	-0.44**	-0.56*	0.49**	-0.42**	-0.31**	1	
Anxiety	0.01	0.05*	0.02	-0.25**	-0.25**	-0.29*	0.47**	-0.16**	-0.19**	-0.23**	1

Note.

*P <0.05,

**P < 0.01.

### Analysis of the chain mediating effect of general self-efficacy and academic burnout

**Fig 1:**
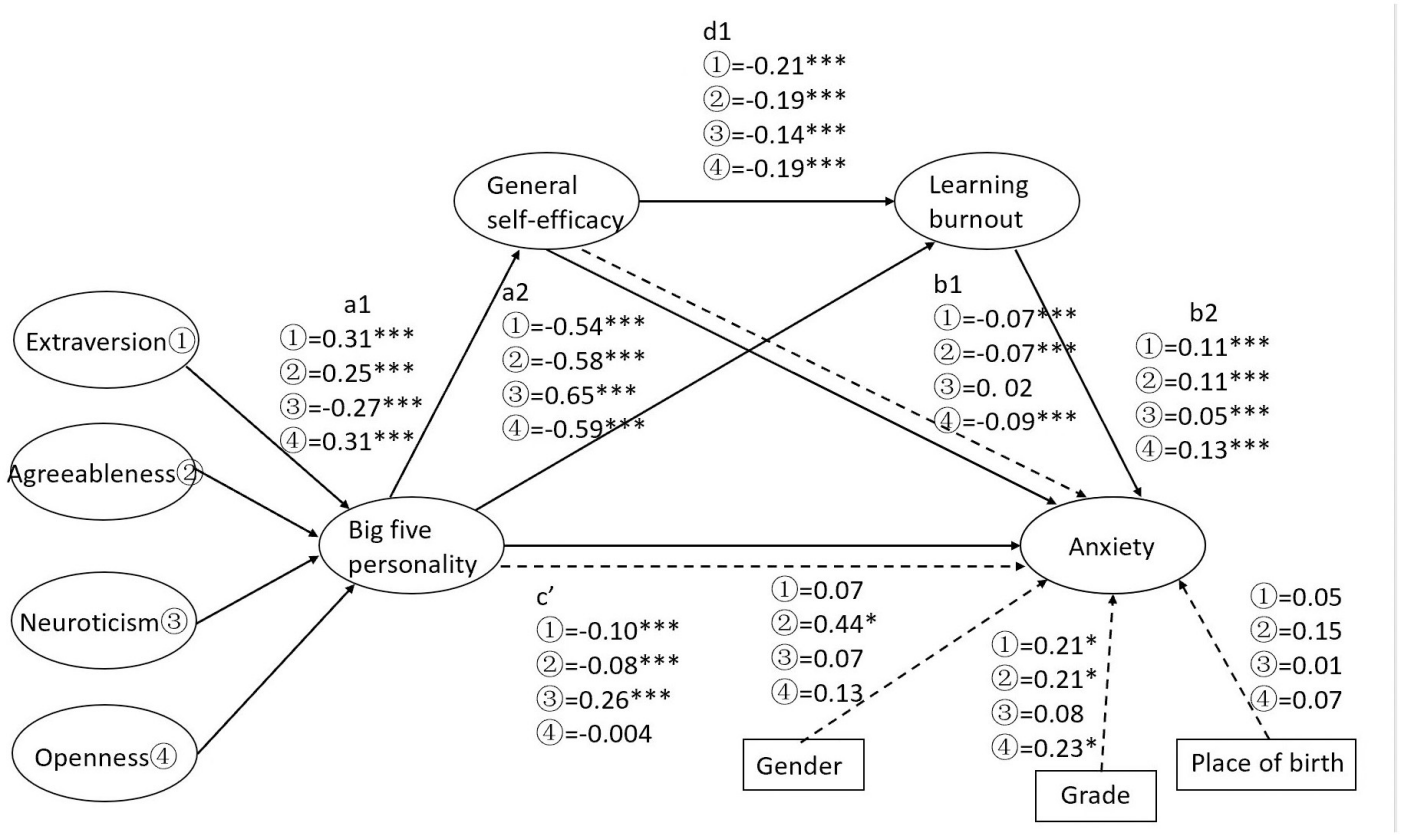
Diagram of the multiple mediating effects of general self-efficacy and academic burnout on the relationship between the Big Five personality and anxiety. Note. *P<0.05, ***P<0.001,a1:Big five personality→General self-efficacy, a2:Big five personality→Learning burnout, b1:General self-efficacy→Anxiety, b2:Learning burnout→Anxiety, c’:Big five personality→Anxiety, d:General self-efficacy→Learning burnout.

This study constructed a structural equation model to test the chain mediating effects of general self-efficacy and academic burnout between the Big Five personality and anxiety. Mediation tests were performed through the SPSS26.0 and PROCESS macro. The results were shown: Extraversion could positively predict general self-efficacy (*β* = 0.31, *P*<0.05) but negatively predict academic burnout (*β* = -0.54, *P*<0.05) and anxiety (*β* = -0.10, *P* <0.05); general self-efficacy could negatively predict academic burnout (*β* = -0.21, *P*<0.05) and anxiety (*β* = -0.07, *P*<0.05); academic burnout could positively predict anxiety (*β* = 0.11, *P*<0.05). The mediating tests showed that general self-efficacy and academic burnout partially mediated the relationship between extraversion and anxiety (the effect value was -0.10, accounting for 46.67% of the total effect). Specifically speaking, the total indirect effect consisted of three paths: the mediating effect of general self-efficacy (the effect value is -0.02, and the effect size is 11.22%); the mediating effect of academic burnout (the effect value is -0.06, and the effect size is 31.74%); the chain mediating effect of general self-efficacy and academic burnout (the effect value is -0.01, and the effect size is 3.76%). The Bootstrap 95%CI of the three indirect effects did not contain 0, indicating that the three indirect effects were all statistically significant. See Table 3, Fig 1.

**Table 3:** The mediating effect analysis of general self-efficacy and academic burnout.

Effect	Path	Effect size	95%CI
Direct effect	Extraversion → Anxiety	-0.10	-0.13~-0.07
	Agreeableness → Anxiety	-0.08	-0.11~-0.05
	Conscientiousness → Anxiety	-0.09	-0.12~-0.06
	Neuroticism → Anxiety	0.26	0.23~0.29
	Openness → Anxiety	-0.01	-0.03~0.03
The mediating effect of general self-efficacy	Extraversion → General Self-efficacy → Anxiety	-0.02	-0.03~-0.01
	Agreeableness → General Self-efficacy → Anxiety	-0.02	-0.03~-0.01
	Conscientiousness → General self-efficacy → Anxiety	-0.02	-0.03~-0.01
	Neuroticism → General self-efficacy →Anxiety	0.01	-0.003~0.02
	Openness → General self-efficacy → Anxiety	-0.03	-0.04~-0.02
The mediating effect of academic burnout	Extraversion → Academic burnout → Anxiety	-0.06	-0.07~-0.05
	Agreeableness → Academic burnout → Anxiety	-0.06	-0.08~-0.05
	Conscientiousness → Academic burnout → Anxiety	-0.08	-0.09~-0.06
	Neuroticism → Academic burnout → Anxiety	0.03	0.02~0.04
	Openness →Academic burnout → Anxiety	-0.08	-0.09~-0.06
Chain mediation effect	Extraversion → General Self-efficacy → Academic burnout → Anxiety	-0.01	-0.01~-0.004
	Agreeableness → General Self-efficacy →Academic burnout → Anxiety	-0.01	-0.01~-0.003
	Conscientiousness → General self-efficacy → Academic burnout → Anxiety	-0.002	-0.004~0.0004
	Neuroticism → General self-efficacy → Academic burnout → Anxiety	0.002	0.001~0.003
	Openness → General self-efficacy → Academic burnout → Anxiety	-0.01	-0.01~-0.004

Agreeableness could positively predict general self-efficacy (*β* = 0.25, *P*<0.05) but negatively predict academic burnout (*β* = -0.58, P<0.05) and anxiety (*β* = -0.08, *P*<0.05); general self-efficacy could negatively predict academic burnout (*β* = -0.19, *P*<0.05) and anxiety (*β* = -0.07, *P*<0.05); academic burnout could positively predict anxiety (*β* = 0.11, *P*<0.05). The results of mediating tests showed that general self-efficacy and academic burnout partially mediated the relationship between agreeableness and anxiety (the effect value was -0.09, accounting for 52.34% of the total effect). Specifically, the total indirect effect consisted of three paths: the mediating effect of general self-efficacy (the effect value is -0.02, and the effect size is 11.41%); the mediating effect of academic burnout (the effect value is -0.06, and the effect size is 37.83%); the chain mediation effect of general self-efficacy and academic burnout (the effect value is -0.01, and the effect size is 3.10%). The Bootstrap 95%CI of the three indirect effects did not contain 0, indicating that the three indirect effects were all statistically significant. See Table 3, Fig 1.

Conscientiousness could positively predict general self-efficacy (*β* = 0.31, *P*<0.05) but negatively predict academic burnout (*β* = -0.78, *P*<0.05) and anxiety (*β* = -0.09, *P*<0.05); general self-efficacy could negatively predict academic burnout (*β* = -0.06, *P*<0.05); academic burnout could positively predict anxiety (*β* = 0.10, *P*<0.05). The results of the mediating tests showed that both general self-efficacy and academic burnout partially mediated the relationship between conscientiousness and anxiety. However, the chain mediating effect of general self-efficacy and academic burnout was insignificant. The Bootstrap 95%CI of the chain mediation effect all contained 0, indicating that the chain mediation indirect effect was not statistically significant. See Table 3, Fig 1.

Neuroticism could positively predict academic burnout (*β* = 0.65, *P*<0.05) and anxiety (*β* = 0.26, *P*<0.05) but negatively predict general self-efficacy (*β* = -0.27, *P*<0.05); General self-efficacy could negatively predict academic burnout (*β* = -0.14, *P*<0.05); academic burnout could positively predict anxiety (*β* = 0.05, *P*<0.05); the mediating role of general self-efficacy was insignificant (*β* = -0.02, *P*>0.05). Though general self-efficacy couldn’t mediate the relationship between neuroticism and anxiety, it could negatively predict academic burnout and positively predict anxiety. In other words, neuroticism could improve general self-efficacy to reduce academic burnout, thus alleviating anxiety. The results of mediating tests showed that general self-efficacy and academic burnout partially mediated between Neuroticism and anxiety (the effect value was 0.04, accounting for 12.79% of the total effect). In addition, the total indirect effect consisted of two paths: the mediating effect of academic burnout (the effect value is 0.03, and the effect size is 10.28%) and the chain mediating effect of general self-efficacy and academic burnout (the effect value is 0.002, the effect size is 0.57%). The Bootstrap 95%CI of the two indirect effects does not contain 0, indicating that both indirect effects were statistically significant. See Table 3, Fig 1.

Openness could positively predict general self-efficacy (*β* = 0.31, *P*<0.05) but negatively predict academic burnout (*β* = -0.59, *P*<0.05); general self-efficacy could negatively predict academic burnout (*β* = -0.19, *P*<0.05) and anxiety (*β* = -0.09, *P*<0.05); academic burnout could positively predict the level of anxiety (*β* = 0.13, *P*<0.05), while the direct association between openness and anxiety was insignificant (*β* = -0.004, *P*>0.05). The results of the mediating effect showed that general self-efficacy and academic burnout completely mediated the relationship between openness and anxiety (the effect value was -0.11, accounting for 96.93% of the total effect). Specifically, the total indirect consisted of three paths: the mediating effect of general self-efficacy (the effect value is -0.03, and the effect size is 24.63%); the mediating effect of academic burnout (the effect value is -0.08, and the effect size is 65.91%); the chain mediation effect of general self-efficacy and academic burnout (the effect value is -0.01, and the effect size is 6.40%). The Bootstrap 95%CI of the three indirect effects does not contain 0, indicating that the three indirect effects are all statistically significant. See Table 3, Fig 1.

## Discussion

The results show that general self-efficacy is negatively correlated with anxiety, while academic burnout is positively correlated with anxiety. General self-efficacy and academic burnout could mediate the association between the big five personality traits and anxiety consistent with prior findings [47–49]. This study focuses on exploring the chain mediating effects of general self-efficacy and academic burnout on the relationship between personality traits and anxiety, as well as examining the direct effects of the five dimensions of the Big Five personality on anxiety. At the same time, in the study of Piepiora et al., it has been pointed out that in health-related conditions, personality traits can affect some unhealthy or addictive physical behaviors in the elderly, and these physical behaviors can lead to the production of anxiety [50]. The results also revealed a robust relationship between Big Five personality and anxiety, which is in line with previous studies [51,52]. The findings also revealed that the chain mediation effect of general self-efficacy and academic burnout on the conscientiousness-anxiety relationship is non-significant, whereas the chain mediation effect of the relationship with the other four personality traits and anxiety are significant. Extraversion, agreeableness, and openness can affect anxiety through three mediating paths: the mediating effect of general self-efficacy; the mediating effect of academic burnout; the chain mediating effect of general self-efficacy and academic burnout. This finding is in line with a study by Alkholy, that found self-efficacy partially mediated the relationship between personality traits (conscientiousness, agreeableness and extraversion) [53]. Neuroticism through two mediating paths affect anxiety: the mediating effect of academic burnout; the chain mediating effect of general self-efficacy and academic burnout. Furthermore, the results also showed that general self-efficacy and academic burnout partially mediated the relationship between personality traits (i.e, extraversion, agreeableness, and neuroticism) and anxiety. In comparison, general self-efficacy and academic burnout completely mediate the relationship between openness and anxiety.

Firstly, this study found the mediating role of general self-efficacy in the association between the five personality traits and anxiety. It suggested that individuals with high level of extraversion, openness, agreeableness, and conscientiousness usually possess high self-efficacy, which can effectively reduce anxiety levels. The finding was consistent with prior research, which proved lower level of neuroticism and higher level of extraversion, openness, agreeableness and conscientiousness was associated with higher level of self-efficacy [21]. And neuroticism had an indirect negative effect on academic performance through self-efficacy [53]. To further explain, positive personality could keep individuals motivated and improve the general self-efficacy by maintaining their core self-evaluation and recognition of their ability at a high level. In addition, self-efficacy was negatively associated with anxiety [54]. Therefore, anxiety reduction could be achieved by improving individual’s general self-efficacy.

Secondly, this study found that academic burnout mediated the relationship between Big Five personality and anxiety, indicating that personality traits could influence the level of anxiety by affecting the sense of academic burnout. Precious studies have shown that personality is a predictor of academic burnout, higher level of extraversion, agreeableness, conscientiousness and openness could predict lower level of academic burnout, while higher level of could predict higher level of academic burnout [48,49]. Specifically speaking, students with higher level of neuroticism have become a high-risk population of academic burnout. They are more likely to experience negative emotions, which could distract students from concentration [55]. Especially during the COVID-19 pandemic, under the background of online learning, the inability to concentrate would increase negative emotions and decrease academic accomplishment, thereby leading to academic burnout [56]. Long-term academic burnout in turn increases the level of anxiety [57]. In conclusion, universities should pay attention to students’ mental health and create a good learning environment for them to alleviate academic burnout and anxiety symptoms.

Thirdly, this study also found that personality traits could affect anxiety levels through the chain mediating effect of general self-efficacy and academic burnout. The chain mediation test was significant in the relationship between personality traits (i.e., extraversion, agreeableness, neuroticism and openness,) and anxiety. Among these personality traits, only openness insignificantly predicted anxiety. That is, general self-efficacy and academic burnout completely mediated the relationship between partially mediated the relationship between personality traits (ie, extraversion, agreeableness, and neuroticism) and anxiety but completely mediated openness personality and anxiety. The findings were consistent with previous research that academic burnout was negatively correlated with general self-efficacy but positively correlated with anxiety [42,58]. In other words, students with high level of extraversion, agreeableness, openness and low level of neuroticism usually possess high level of general self-efficacy. If a student recognized his ability and developed a higher sense of accomplishment, then it would be easier for him to keep motivated and maintain interests in learning, which accounted for less academic burnout and anxiety.

## Limitations and future directions

All samples were from the same university. The homogeneity of the participants could not represent all the college students in China.

A larger sample size should be studied in the future. In addition, this study aims at exploring the chain mediating effect of general self-efficacy and academic burnout, so many other factors might be ignored. In the future, more influencing factors should be introduced to abundant the research on anxiety, and deeper analysis is needed. Beyond that, the cross-sectional design of the original study makes it difficult to reveal the causal relationship between personality traits and anxiety. Follow-up studies could apply the longitude method for further verification.

## Conclusions

1. There is a high correlation between personality traits (i.e., extraversion, agreeableness, neuroticism, and openness) and anxiety.
2. General self-efficacy negatively predicted anxiety level, while academic burnout positively predicted anxiety level.
3. Personality traits can indirectly influence anxiety through the chain mediating effects of general self-efficacy and academic burnout.

## Supporting information

S1 Data (XLS)
